# Critical role of Znhit1 for postnatal heart function and vacuolar cardiomyopathy

**DOI:** 10.1172/jci.insight.148752

**Published:** 2022-03-22

**Authors:** Yingchao Shi, Wenli Fan, Mingjie Xu, Xinhua Lin, Wukui Zhao, Zhongzhou Yang

**Affiliations:** 1 State Key Laboratory of Pharmaceutical Biotechnology, MOE Key Laboratory of Model Animal for Disease Study, Model Animal Research Center, and Jiangsu Key Laboratory of Molecular Medicine, Nanjing University Medical School, Nanjing, China.; 2 State Key Laboratory of Genetic Engineering, School of Life Sciences, Zhongshan Hospital, Fudan University, Shanghai, China.

**Keywords:** Cardiology, Calcium, Cardiovascular disease, Heart failure

## Abstract

Ca^2+^ is critical for cardiac electrical conduction and contractility, and aberrant Ca^2+^ homeostasis causes arrhythmia and heart failure. Chromatin remodeling modulates gene expression involved in cardiac sarcomere assembly and postnatal heart function. However, the chromatin-remodeling regulatory mechanism of cardiac Ca^2+^ homeostasis is unknown. Here, we found that Znhit1, a core subunit of the SRCAP remodeling complex, was essential for heart function. Deletion of *Znhit1* in postnatal hearts of mice resulted in arrhythmia, idiopathic vacuolar cardiomyopathy, rapid heart failure, and premature sudden death. In addition, the level of Casq1, a sarcoplasmic reticulum Ca^2+^ regulatory protein, was massively elevated while SERCA2a showed reduced protein level. Mechanistically, the Znhit1 modulated the expression of *Casq1* and *SERCA2a* by depositing H2A.Z at their promoters. Deletion of *Casq1* could substantially alleviate the vacuolar formation in *Znhit1* *Casq1* KO mice. These findings demonstrate that Znhit1 is required for postnatal heart function and maintains cardiac Ca^2+^ homeostasis and that accumulation of Casq1 might be a causative factor for vacuolar cardiomyopathy.

## Introduction

Ca^2+^ is critical for cardiac electrical conduction and contraction (1, 2). While excitation-contraction coupling triggers Ca^2+^ release from the sarcoplasmic reticulum (SR) to cytoplasm via ryanodine receptors (RyRs), uptake of Ca^2+^ back to the SR is largely accomplished by SR Ca^2+^-ATPase 2a (SERCA2a) (2, 3). In the SR, Ca^2+^ is retained by the most abundant Ca^2+^-binding protein, calsequestrin 2 (Casq2) (4). Casq1 is highly homologous to Casq2 and these 2 proteins function similarly to regulate Ca^2+^ homeostasis in muscle cells (5). Although both Casq1 and Casq2 are present in skeletal muscles, only Casq2 is found in cardiomyocytes. Mouse genetics studies demonstrated that loss of either *Casq1* or *Casq2* failed to cause lethal cardiomyopathy, although SR Ca^2+^ homeostatic regulation was disrupted (5). On the contrary, transgenic mice with *Casq2* overexpression in cardiomyocytes had severe dilated cardiomyopathy and died prematurely by age 16 weeks (6, 7).

Vacuolar cardiomyopathy is a rare but lethal heart disease with features of prominent vacuoles in the myofiber. It is usually associated with lysosomal functional deficiencies, including storage disorders (namely, Pompe disease, resulting from acid α-glucosidase deficiency) and protein deficiency (namely, Danon disease, resulting from LAMP2 deficiency) (8–10). However, non–lysosome-related vacuolar cardiopathy was frequently observed and its pathogenesis needs investigation (11, 12).

The chromatin-remodeling complexes regulate massive gene expression (13). Previously, it was reported that the SWI/SNF chromatin-remodeling complex modulates heart development and postnatal heart growth (14). For instance, Brg1, a core component of the SWI/SNF chromatin remodeler, promotes embryonic cardiomyocyte proliferation and preserves cardiac differentiation (15). In adult mice, cardiac stress–activated Brg1 induces a pathological α-MHC to β-MHC shift that leads to hypertrophy (15). In addition to the SWI/SNF chromatin-remodeling complex, 3 other chromatin remodelers (ISWI, NuRD, and INO80/SWR complex) exist in mammals (13). However, compared with the SWI/SNF complex, the functions of these 3 chromatin-remodeling complexes in the postnatal heart remain unknown.

The zinc finger HIT-domain–containing protein 1 (Znhit1; Supplemental Table 1; supplemental material available online with this article; https://doi.org/10.1172/jci.insight.148752DS1), is a key subunit of the INO80/SWR chromatin-remodeling complex (also called SRCAP chromatin-remodeling complex), which regulates gene transcription by depositing histone variant H2A.Z at promoters (16). Recently, authors of several studies have reported the function of SRCAP/Znhit1 in maintaining stemness and fate determination of stem cells, such as hematopoietic stem cells, intestinal stem cells, and lymphoid lineage commitment (17–19).

In the present study, we aimed to understand the role of Znhit1 in the postnatal heart. We generated cardiomyocyte-specific *Znhit1*-deletion mice, which developed arrhythmia, idiopathic vacuolar cardiomyopathy, and rapid heart failure leading to premature sudden death. Additionally, Znhit1 deficiency massively elevated Casq1 levels, and deletion of *Casq1* could substantially alleviate the vacuolar formation in *Znhit1* *Casq1* KO (cKO) mice. Thus, we determined a function of the SRCAP chromatin remodeler in regulating Ca^2+^ homeostasis and heart function. Our findings indicate a causative mechanism of non–lysosomal-related vacuolar myopathy.

## Results

### Deletion of Znhit1 caused premature sudden death and pathological heart remodeling.

**Figure 1:**
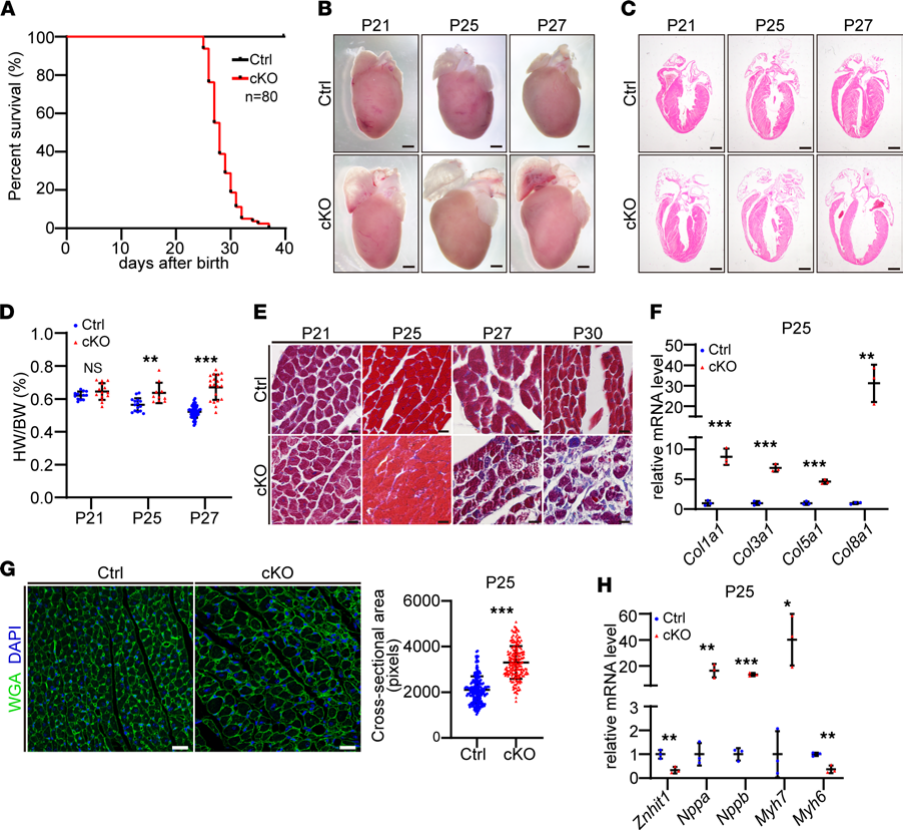
Deletion of Znhit1 caused premature sudden death and pathological cardiomyopathy. (**A**) Survival curves of cKO mice (*n* = 80) and littermate controls (Ctrls). (**B**) Representative images of mouse hearts. Scale bars: 1 mm. (**C**) Representative images of H&E staining of the heart. Scale bar: 1 mm. (**D**) Ratio of heart weight (HW) to BW. Numbers of mice were as follows: Ctrl: on P21 (*n* = 12), P25 (*n* = 15), and P27 (*n* = 52); cKO: on P21 (*n* = 14), P25 (*n* = 10), and P27 (*n* = 20). (**E**) Representative Masson’s trichrome staining of heart tissues. Scale bar: 10 μm. (**F**) mRNA expression levels of fibrosis-related gene. *n* = 3 for each group. (**G**) Representative images of WGA IF staining (left). Scale bar: 20 μm. Quantitative analysis of cardiomyocytes’ cross-sectional area (right) (Ctrl, *n* = 196; cKO, *n* = 180). (**H**) qRT-PCR analysis of cardiac hypertrophic marker genes. *n* = 3 for each group. Ctrl mice were *Znhit1^fl/fl^* or *Znhit1^fl/+^* littermates. Data are presented as mean ± SD. *P < 0.05, **P < 0.01, and ***P < 0.001 by unpaired 2-tailed Student’s *t* test.

Previous studies indicate that disruption of Znhit1, a core subunit of the SRCAP chromatin-remodeling complex, greatly impairs the assembly of SRCAP complex and function (17, 19). To understand the function of the SRCAP chromatin-remodeling complex in the postnatal heart, we deleted *Znhit1* specifically in postnatal cardiomyocytes by breeding *Znhit1* floxed (*Znhit1^fl/fl^*) mice with *Myh6-Cre* mice (20, 21) to generate *Znhit1^fl/fl^; Myh6-Cre* mice (hereafter referred to as *Znhit1* cKO). The efficiency of *Znhit1* deletion was determined by quantitative real-time PCR (qRT-PCR) and Western blotting using heart tissues from mice on P10 (Supplemental Figure 1, A–C). Sudden death of the *Znhit1* cKO mice was observed on P25, and a majority of the cKO mice (>81.25%) died between P25 and P30 (Figure 1A). None of the cKO mice survived beyond 40 days (Figure 1A). Compared with littermate controls, the *Znhit1* cKO mice did not exhibit any obvious differences in BW before P25 (Supplemental Figure 1D). However, the BW of the *Znhit1* cKO mice was markedly reduced on P27, suggesting slowed growth by this stage (Supplemental Figure 1D). The temporal expression patterns of *Znhit1* in the heart of WT mice were determined by qRT-PCR, and the results demonstrated that the expression level of *Znhit1* decreased steadily after birth (Supplemental Figure 1, F and G). At P25, the expression level of *Znhit1* did not show a big change, excluding the possibility that P25 was a critical time point for Znhit1 to function in the heart (Supplemental Figure 1, F and G).

Subsequently, we studied the phenotypic alterations of *Znhit1* cKO hearts by histological analysis. While only right atrium dilation was observed on P21, it was obvious that the heart became bigger and the right atrium was dramatically dilated with thinned walls on P25 and P27, compared with the control hearts (Figure 1, B and C). Meanwhile, dilation of the left ventricular chamber was also noticeable on P27 (Figure 1C). The hearts of the *Znhit1* cKO mice exhibited substantially increased mass compared with control hearts (Supplemental Figure 1E). Accordingly, the ratio of heart weight to BW was significantly augmented in *Znhit1* cKO mice on P25 and P27 (Figure 1D). In addition, Masson’s trichrome staining indicated that cardiac interstitial fibrosis was apparent on P25 and more obvious on P27 and P30 (Figure 1E). qRT-PCR analysis showed strong upregulation of fibrosis-related genes, including *Col1a1*, *Col3a1*, *Col5a1*, and *Col8a1* in cKO hearts (Figure 1F). Immunofluorescence (IF) staining of wheat germ agglutinin (WGA) revealed profoundly enlarged cardiomyocyte area in cKO mice on P25 (Figure 1G). mRNA expression of cardiac hypertrophy marker genes *Nppa, Nppb, Myh6*, *and Myh7* was massively enhanced compared with genes in control mice on P25 (Figure 1H). However, no apoptotic cardiomyocytes were observed between P25 and P27 by TUNEL staining (Supplemental Figure 1H). Collectively, these results indicate that loss of Znhit1 in the postnatal heart causes pathological cardiomyopathy leading to premature sudden death.

### Rapid heart failure and arrhythmia in Znhit1 cKO mice.

**Figure 2:**
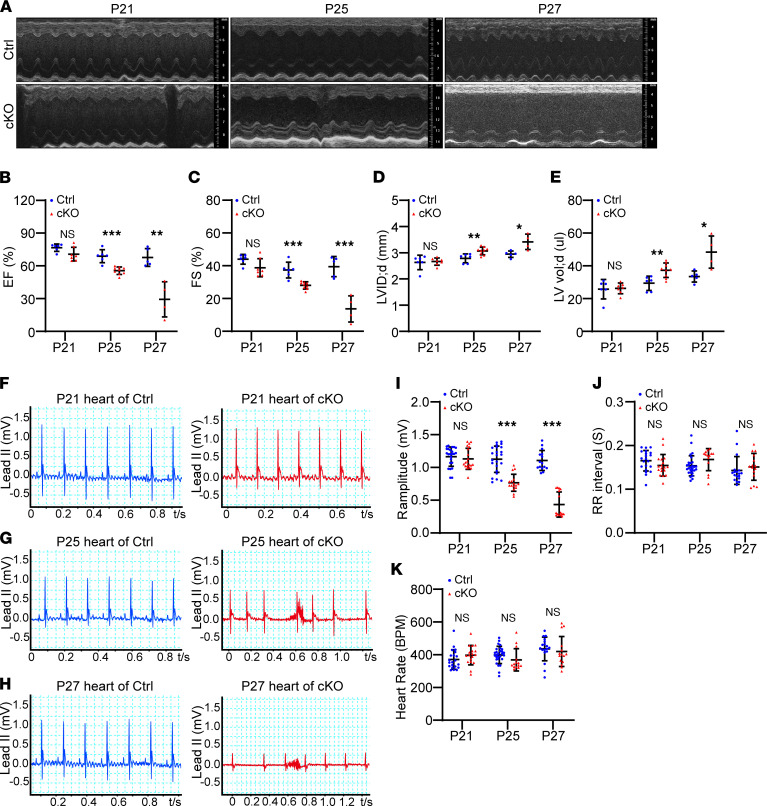
Znhit1 cKO mice had rapid heart failure and arrhythmia. (**A**) Representative Echo images. (**B **and** C**) Quantitative analysis of ejection fraction (EF) and fractional shortening (FS). Ctrl, P21 (*n* = 6); P25 (*n* = 6); P27 (*n* = 5). cKO, P21 (*n* = 7); P25 (*n* = 9); P27 (*n* = 4). (**D**) Quantitative analysis of left ventricular internal dimension at diastole (LVID; d). (**E**) Quantitative analysis of left ventricular volume at diastole (LV vol; d). Numbers of mice were as follows: control (Ctrl), on P21 (*n* = 6), P25 (*n* = 6), and P27 (*n* = 5); cKO: on P21 (*n* = 7), P25 (*n* = 9), and P27 (*n* = 4). (**F–H**) ECG: left panels, Ctrl; right panels, Znhit1 cKO. (**I**) Quantitative analysis of R amplitude. Numbers of mice were as follows: Ctrl, on P21 (*n* = 24), P25 (*n* = 23), and P27 (*n* = 15); and cKO: on P21 (*n* = 20), P25 (*n* = 16), and P27 (*n* = 15). (**J **and** K**) Quantitative analysis of the RR interval and heart rate in Ctrl and Znhit1 cKO mice. Numbers of mice were as follows: Ctrl, on P21 (*n* = 21), P25 (*n* = 28), and P27 (*n* = 18); and cKO: on P21 (*n* = 18), P25 (*n* = 14), and P27 (*n* = 15). Ctrl mice were *Znhit1^fl/fl^* or *Znhit1^fl/+^* littermates. Data are presented as mean ± SD. *P < 0.05, **P < 0.01, and ***P < 0.001 by unpaired 2-tailed Student’s *t* test.

Next, we used echocardiography (Echo) and ECG to evaluate cardiac function and cardiac electrical activity in control and *Znhit1* cKO mice. Echo showed that cardiac function was nearly normal in cKO mice on P21 (Figure 2, A–C); however, heart contraction became abnormal, and substantially decreased cardiac systolic function was observed in the cKO mice on P25, which was worsened on P27 (Figure 2, A–C). Meanwhile, the other 2 cardiac parameters—diastolic left ventricular internal diameter and diastolic left ventricular volume—of cKO mice were significantly greater than that of control mice on P25 (Figure 2, D and E), which indicated ventricular dilation. The left ventricle (LV) of cKO mice was further enlarged on P27 (Figure 2, D and E). These Echo results revealed a rapid heart failure in *Znhit1* cKO mice. We also measured the thickness of the LV wall of control and cKO mice and found reduced LV wall thickness in the cKO mice compared with control mice (Supplemental Figure 2A).

Consistently, ECG exhibited prominent abnormalities with the R wave amplitude and the RR intervals (Figure 2, F–H). At P21, the RR intervals and heart rates were comparable between cKO and control mice (Figure 2F). However, on P25 and P27, decreased R wave amplitude and irregular RR intervals were apparent in the cKO mice (Figure 2, G and H). Quantitative analysis results for R wave amplitude, heart rate, and RR interval are shown in Figure 2, I–K. These changes in the cKO mice manifested the features of atrial fibrillation and/or atrial standstill. Taken together, these studies demonstrated that Znhit1 deficiency resulted in rapid heart failure and arrhythmia, leading to premature sudden death in mice.

### Altered expression pattern of SR Ca^+^ handling proteins and SR Ca^+^ homeostasis.

The SRCAP complex regulates gene expression; therefore, we performed RNA-Seq to explore the transcriptomic difference between *Znhit1* cKO and control hearts. For this purpose, heart tissues were harvested from mice on P17 and P25. At P17, pathological cardiac remodeling in cKO mice was not discernible compared with on P25, as indicated by expression levels of pathological remodeling genes (Supplemental Figure 3, A and B, and Figure 1, F and H). Thus, the time points of P17 and P25 represented the pathological alteration from the early stage to mid to late stage.

At P17, genes with | log2(fold change) | ≥ 1 and *P* ≤ 0.05 were considered significantly different between cKO and control mice. A total of 472 differentially expressed genes were identified, 260 of which were downregulated and 212 were upregulated (Supplemental Figure 3C). The small number of differentially expressed genes manifested the early features of *Znhit1* cKO mice on P17, which made it difficult to perform the cluster analysis. To facilitate data analysis, genes with | log2(fold change) | ≥ 0.58 and *P* ≤ 0.05 were regarded as significantly different. A total of 1135 differentially expressed genes were identified, 567 of which were downregulated and 568 were upregulated (Supplemental Figure 3D). The up- and downregulated genes are displayed with heatmaps and volcano plots (Supplemental Figure 3, E and F). At P25, genes with | log2(fold change) | ≥ 1 and *P* ≤ 0.05 were considered significantly different between cKO and control mice. A total of 3417 differentially expressed genes were identified, 1694 of which were downregulated and the 1723 upregulated (Supplemental Figure 3, G–I).

**Figure 3:**
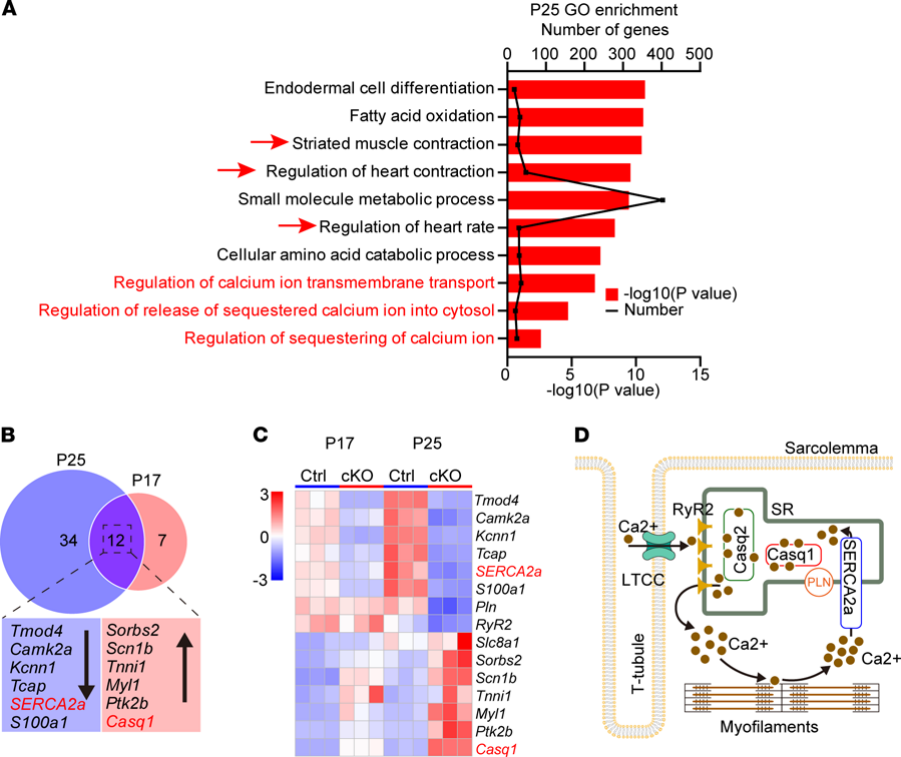
Transcriptomic profiling of hearts tissues. (**A**) GO analysis of differentially expressed genes on P25. (**B**) Overlapping of differentially expressed genes at both P17 and P25. Downward-pointing black arrows indicate downregulated genes; upward-pointing arrows indicate upregulated genes. (**C**) Heatmap analysis. Ca^2+^ handling genes (*SERCA2A* and *Casq1*) are indicated by red. (**D**) Diagram showing Ca^2+^-handling proteins, Ca^2+^ storage, and release in the SR of cardiomyocytes. Under normal conditions, Casq2 is abundant and a little Casq1 is present in the SR. PLN, phospholamban; RyR2, ryanodine receptor 2; LTTC, L-type calcium channel. Control (Ctrl) mice were *Znhit1^fl/fl^* or *Znhit1^fl/+^* littermates.

Next, we performed gene ontology (GO) analysis of the differentially expressed genes. Genes associated with striated muscle contraction, heart contraction, and heart rate were enriched from GO analysis of P25 (Figure 3A). Specific to our interest, calcium-handling genes were present in the enrichment and were involved in SR Ca^+^ homeostatic regulation (Figure 3A).

**Figure 4:**
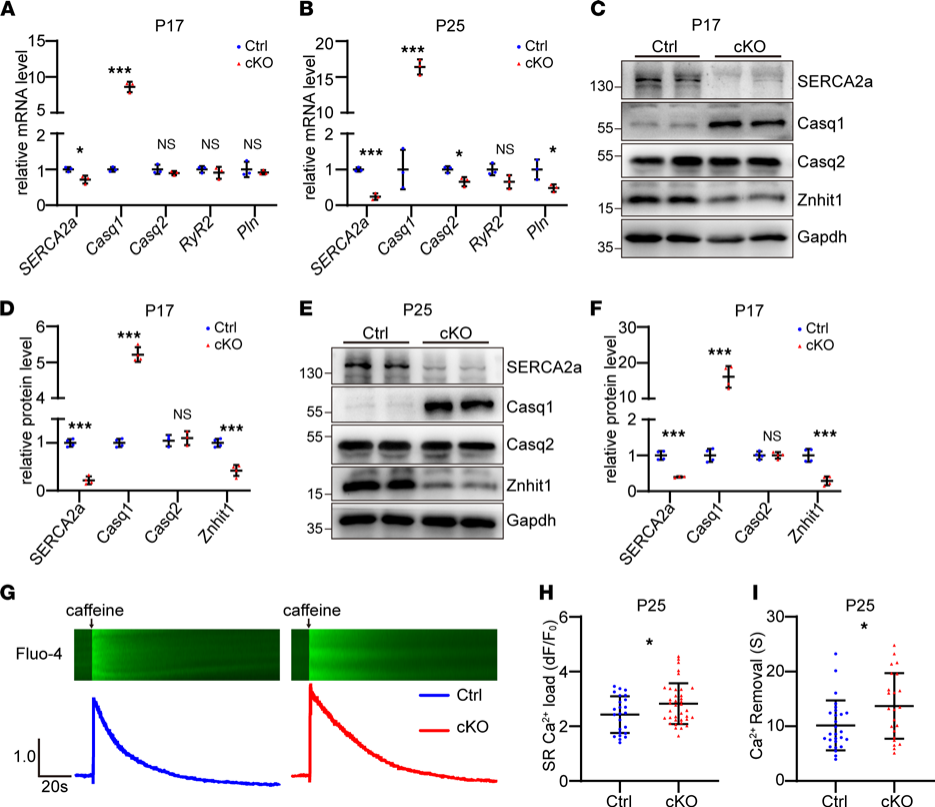
Altered expression pattern of SR Ca^+^-handling proteins and SR Ca^+^ homeostasis. (**A **and** B**) qRT-PCR analysis. *n* = 3 for each group. (**C **and** D**) Western blotting and quantitative analysis on P17. Gapdh was used as a loading control. (**E **and** F**) Western blotting and quantitative analysis on P25. Gapdh was used as a loading control. (**G**) Representative images of caffeine-induced calcium transients with Fluo-4 dye. F, fluorescence signal intensity; F_0_, basal signal intensity; dF = F – F_0_. (**H**) Quantitative analysis of the amplitudes (dF/F_0_) of caffeine-induced Ca^2+^ signal. Ctrl (*n* = 24) and cKO (*n* = 37). (**I**) Quantitative analysis of the decay time of calcium signal. Ctrl (*n* = 27) and cKO (*n* = 23). Control (Ctrl) mice were *Znhit1^fl/fl^* or *Znhit1^fl/+^* littermates. Data are presented as mean ± SD. *P < 0.05, ***P < 0.001 by unpaired 2-tailed Student’s *t* test.

We next focused on studying Ca^+^ regulation by analyzing the differentially expressed genes on P17 an P25. The altered expression pattern of Ca^+^ regulation genes is shown in Supplemental Figure 4, A and B. Of these genes, 12 genes of similar pattern at both P17 and P25 (Supplemental Tables 3 and 4) were identified, 6 of which were upregulated and the other 6 were downregulated (Supplemental Figure 4C and Figure 3, B–D). Further validation of these genes by qRT-PCR confirmed 2 genes with significantly changed expression level: *SERCA2a* and *Casq1* (Figure 4, A and B). Western blotting also verified the markedly reduced protein level of SERCA2a and profoundly enhanced protein level of Casq1 in cKO mice compared with control mice (Figure 4, C and D), which became more evident on P25 (Figure 4, E and F).

SERCA2a facilitates Ca^+^ uptake by SR, and Casq1 retains Ca^+^ inside the SR. To test whether SR Ca^+^ homeostasis was changed, we performed a caffeine-induced Ca^2+^-release experiment in primary cardiomyocytes isolated from P25 mouse hearts. Compared with the control group, the amplitudes of calcium transient were significantly increased, indicating the augmented SR Ca^2+^ storage capacity in *Znhit1* cKO mice (Figure 4, G and H). Meanwhile, the decay time of the calcium signal became longer, suggesting the removal of cytosolic Ca^2+^ was slowed (Figure 4I). Collectively, these data demonstrated altered Ca^2+^ homeostasis in the cKO heart.

### Idiopathic vacuolar cardiomyopathy in Znhit1 cKO mice.

**Figure 5:**
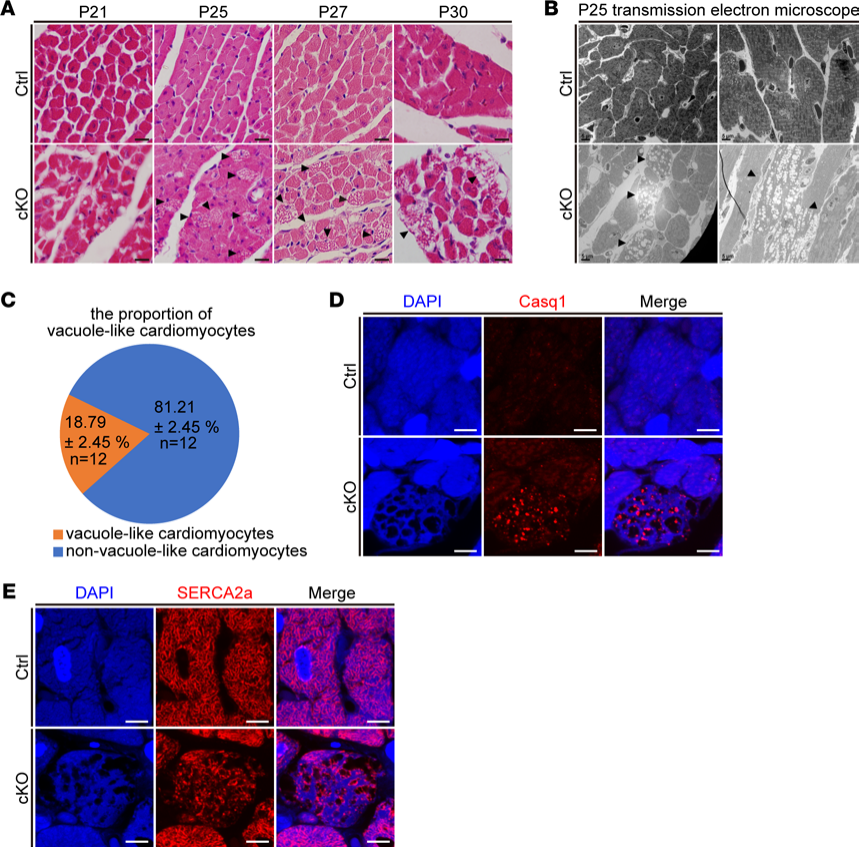
Idiopathic vacuolar cardiomyopathy in *Znhit1* cKO mice. (**A**) H&E staining of heart tissues. Black arrowheads indicate the vacuoles in the cardiomyocytes. Original magnification, ×40. Scale bars: 10 μm. (**B**) TEM imaging of the vacuole-like cardiomyocytes. Black arrowheads indicate the vacuoles in the cardiomyocytes. Original magnification, ×2000. Scale bars: 5 μm. (**C**) The percentage of vacuole-like and non–vacuole-like cardiomyocytes in *Znhit1*-deficient hearts. Two to three sections from 5 hearts of both control (Ctrl) and *Znhit1* mutant mice were quantified. (**D**) Representative IF staining for Casq1. Original magnification, ×100. Scale bars: 5 μm. (**E**) Representative IF staining for SERCA2a. Original magnification, ×100. Scale bars: 5 μm. Ctrl mice were *Znhit1^fl/fl^* or *Znhit1^fl/+^* littermates. Data are presented as mean ± SD.

When performing histological studies of *Znhit1* cKO heart tissues, we observed the feature of progressive vacuolar cardiomyopathy (Figure 5A). At P25, vacuoles were apparent inside the myofibers, and this phenomenon became more evident on P27 (Figure 5A). Transmission electron microscope (TEM) imaging revealed many low-electron-density vacuoles between myocardial fibers (Figure 5B). Quantitative analysis demonstrated approximately 20% of all the cardiomyocytes on P25 had vacuoles in cKO mice (Figure 5C). Careful and detailed study with higher magnification of TEM imaging displayed membrane structure around the empty vacuoles (Supplemental Figure 5A).

Mutant Casq1 in human patients has been linked to vacuolar myopathy (22, 23). To determine whether these vacuoles were associated with Casq1, IF staining for Casq1 was performed and the results revealed Casq1 presence in the vacuoles (Figure 5D). Casq1/2 proteins are localized mainly in the SR terminal cisternae lumen (24–26). Thus, to determine whether these vacuoles were linked with SR, IF staining for SERCA2a was performed and we observed apparently decreased SERCA2a with a disarrayed pattern in the vacuoles (Figure 5E). We also observed vacuoles containing electron-dense materials (Supplemental Figure 5A).

Vacuolar cardiomyopathy has been connected to autophagic and lysosomal dysfunction (27–30). However, we did not observe in the TEM images any accumulation of autophagosomes and autolysosomes containing undigested cytoplasmic materials (Supplemental Figure 5B and Figure 5B). Thus, we thought these vacuoles might be unrelated to autophagosomes and lysosomes. On the other hand, we did not observe any membrane structure of mitochondria in the vacuoles (Supplemental Figure 5, A and B). In addition, the mitochondria surrounding the vacuoles had normal morphology in the cKO cardiomyocytes (Supplemental Figure 5C). Taken together, these results demonstrated an idiopathic vacuolar cardiomyopathy in the *Znhit1* cKO heart.

### Znhit1 deficiency affected the deposition of histone variant H2A.Z at gene promoters.

The SRCAP complex regulates gene transcription by replacing histone H2A with a variant, H2A.Z, at gene promoter regions (31–33). Deletion of *Znhit1* has also been reported to affect the incorporation of H2A.Z in the genome and change the gene transcription levels (17, 19, 34, 35).

**Figure 6:**
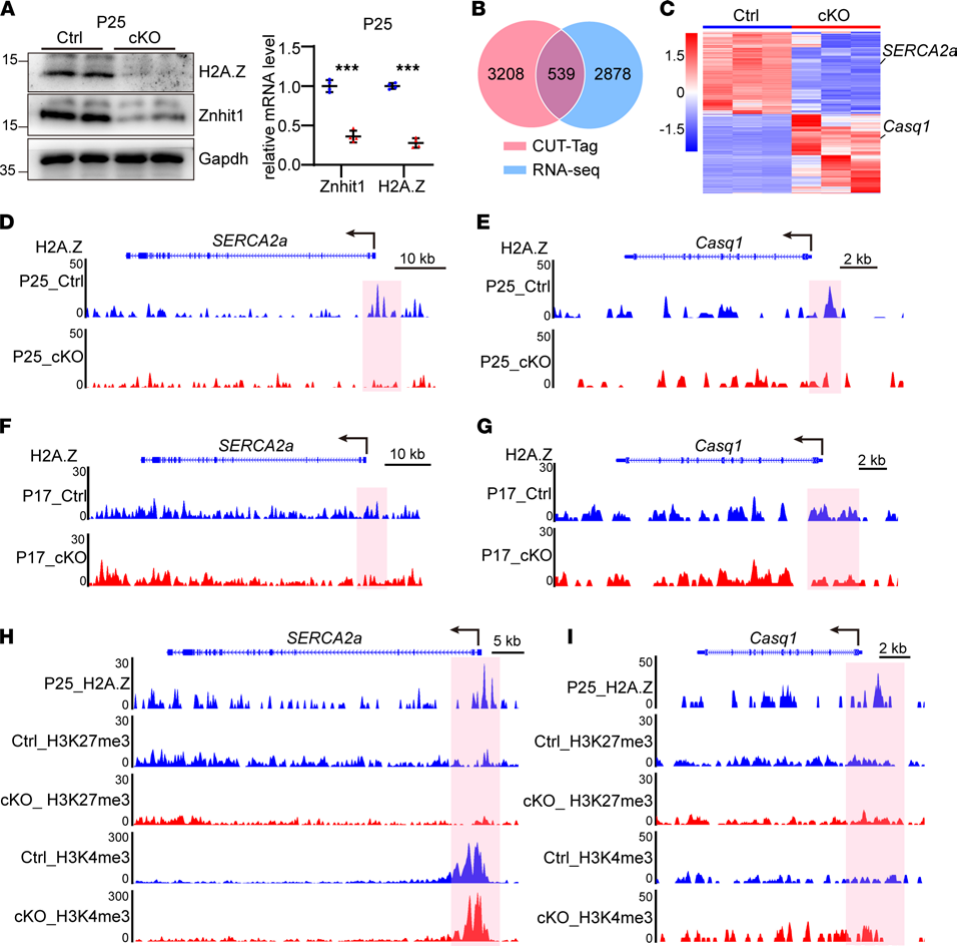
Znhit1-deficiency impaired the deposition of H2A.Z at gene promoters. (**A**) Western blotting and quantitative analysis of H2A.Z on P25. Gapdh was used as a loading control. (**B**) Comparison analysis of the CUT&Tag data and RNA-Seq data on P25. (**C**) Heatmap of the 539 genes in **B**. (**D–G**) The distribution of H2A.Z at the promoter of *SERCA2a* and *Casq1* on P25 and P17. (**H **and** I**) The distribution of H3K27me3 and H3K4me3 at the promoter of *SERCA2a* and *Casq1*. Control (Ctrl) mice were *Znhit1^fl/fl^* or *Znhit1^fl/+^* littermates. Data are presented as mean ± SD. ***P < 0.001 by unpaired 2-tailed Student’s *t* test.

First, we examined the protein levels of H2A.Z and found a substantial reduction of H2A.Z protein in the cKO hearts on P25 (Figure 6A). To test whether the changes in *Casq1* and *SERCA2a* were mediated by impairing the distribution of H2A.Z in the *Znhit1*-deficient mouse hearts, we performed the cleavage under targets and tagmentation (CUT&Tag) experiment with H2A.Z Ab using heart tissues from P25 mice. Compared with traditional ChIP-Seq experiments, CUT&Tag exhibited better repeatability and signal-to-noise ratio with lower cell numbers (36). By analyzing the CUT&Tag results of control heart tissues, we obtained 3747 genes with H2A.Z enrichment at their promoters. Through comparison analysis of the CUT&Tag data and RNA-Seq data (both using heart tissues from P25 mice), we identified 539 differentially expressed genes between control and *Znhit1* cKO mice (Figure 6B). The intensities of H2A.Z enrichment at these gene promoters significantly decreased in *Znhit1*-deficient heart tissues (Supplemental Figure 6A). Among these genes, 273 were downregulated and the other 266 were upregulated (Figure 6C). Kyoto Encyclopedia of Genes and Genomes pathway analysis indicated that Znhit1 deficiency could affect several important biological functions, such as fatty acid metabolism, cardiac muscle contraction, dilated cardiomyopathy, and branched-chain amino acids (including valine, leucine, and isoleucine) degradation (Supplemental Figure 6B).

As expected, the deletion of *Znhit1* decreased significantly the deposition of H2A.Z at the promoter of *Casq1* and *SERCA2a* (Figure 6, D and E). Furthermore, we performed extra CUT&Tag experiments with the Abs of H2A.Z, H3K27me3, and H3K4me3 using heart tissues from P17 mice (Figure 6, F–I). Similar to P25 mice, the enrichment of H2A.Z at the promoter of *SERCA2a* and *Casq1* was profoundly reduced on P17 (Figure 6, F and G). Previous studies reported that reduction of H2A.Z impaired the distribution of H3K4me3 and H3K27me3 (17). Thus, we wanted to know whether the deletion of Znhit1 has a similar effect. We found that at the promoter of *SERCA2a*, H3K4me3, was significantly enriched while H3K27me3 was only slightly enriched. The decline of H2A.Z (upon Znhit1 deletion) mildly decreased the distribution of H3K4me3 and H3K27me3 at the promoter of *SERCA2a* (Figure 6H). On the other hand, we found that at the promoter of *Casq1*, there was almost no enrichment of either H3K4me3 or H3K27me3, and the decline of H2A.Z did not change the distribution of H3K27me3 but slightly increased H3K4me3 enrichment (Figure 6I). Based on these results, we think that the H2A.Z-regulatory mechanisms on these 2 genes might depend on the balance of other activated or repressed conditions. We found no enrichment of H2A.Z at the promoter of *Casq2* (Supplemental Figure 6C).

It has been noted that pathological cardiac remodeling causes the reactivation (i.e., expression) of a panel of fetal genes, including *Myh7*, *Nppa* (*ANP*), and *Nppb* (*BNP*) but causes suppression of *Myh6* (37). Therefore, examination of the expression levels of these genes can distinguish pathological heart remodeling. Previous studies indicated that fetal gene reactivation was regulated by epigenetic modification at different molecular levels, such as DNA methylation, histone modification, and ATP-dependent chromatin remodeling (38). Among them, BAF complex, a member of ATP-dependent chromatin-remodeling complexes SWI/SNF family, has been studied extensively in cardiac development and pathological heart remodeling. The BAF complex regulates the coordinated expression of *Myh6* and *Myh7* (15). To address whether Znhit1 participates in regulating expression of these fetal genes, we compared the H2A.Z distribution at the promoters of these fetal genes by analyzing the CUT&Tag data.

H2A.Z was enriched significantly at the promoters of *Myh6* and *Myh7* in the heart on P25, but not at the *Nppa* and *Nppb* promoters. Upon the deletion of *Znhit1*, the enrichment of H2A.Z was decreased, indicating that Znhit1 was involved in the deposition of H2A.Z at the promoters of *Myh6* and *Myh7* to regulate the expression of these genes (Supplemental Figure 6, E and F).

### Deletion of Casq1 alleviated the vacuolar formation in Znhit1-deficient hearts.

As mentioned, the increased expression of Casq1 might play critical roles in vacuolar formation in *Znhit1*-deficient hearts. To verify this, we generated *Casq1* KO (*Casq1*^–/–^) mice. Western blot analysis confirmed successful ablation of *Casq1* (Supplemental Figure 7, A and B). These mice could survive to adulthood, and histological study of the skeletal muscle and heart of the *Casq1*^–/–^ mice revealed comparable morphology and structure to those of control mice (Supplemental Figure 7, C and D).

**Figure 7:**
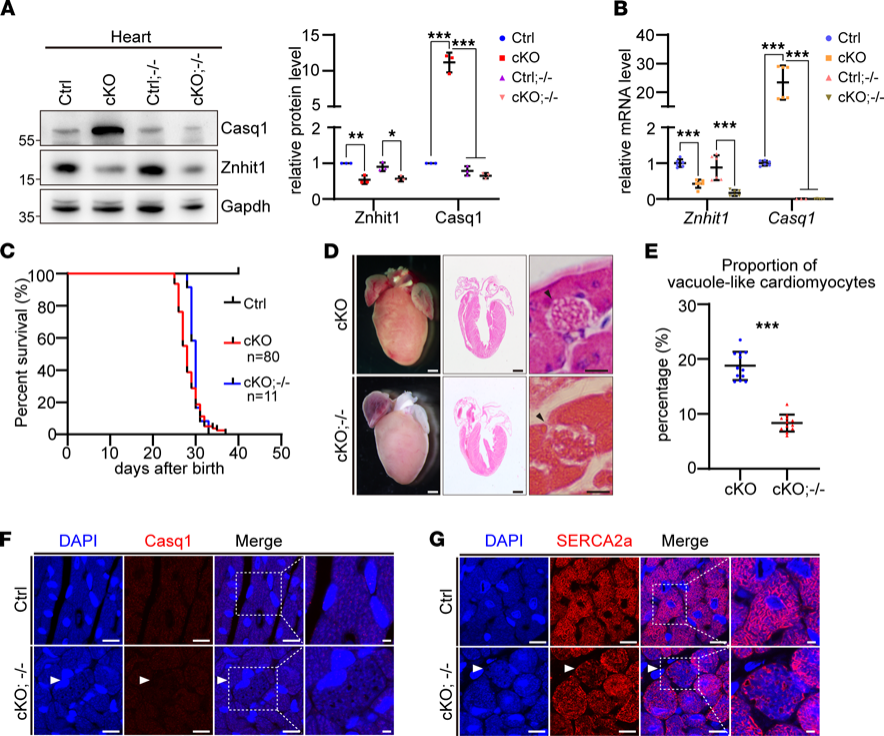
The deletion of *Casq1* in *Znhit1* cKO partially rescued the phenotype of vacuolar formation. (**A **and** B**) Western blotting and quantitative analysis of Casq1 in P25 *Znhit1* cKO; *Casq1*^–/–^ mice. Gapdh was used as a loading control. (**C**) Survival curves of *Znhit1* cKO; *Casq1*^–/–^ mice (*n* = 11) and *Znhit1* cKO mice (*n* = 80). (**D**) The comparison of *Znhit1* cKO and *Znhit1* cKO; *Casq1*^–/–^ mouse heart sections with similar heart morphology. Black arrowheads indicate the vacuole-like cardiomyocytes. Scale bars: 1 mm (left and middle columns), and 20 μm (right column). (**E**) Quantitative analysis of the ratio of vacuole-like cardiomyocytes in P25 *Znhit1* cKO and *Znhit1* cKO; *Casq1*^–/–^ mice. The sample number is at least 3 for each group containing at least 9 sections. (**F **and** G**) Representative IF staining for Casq1 and SERCA2a. Original magnification, ×100. Scale bars: 5 μm. Control (Ctrl) mice were *Znhit1^fl/fl^* or *Znhit1^fl/+^* littermates. Data are presented as mean ± SD. *P < 0.05, **P < 0.01, and ***P < 0.001 by unpaired 2-tailed Student’s *t* test.

Afterward, we crossed *Casq1*^–/–^ mice with cardiomyocyte-specific *Znhit1*-deletion mice (*Znhit1* cKO) to generate *Znhit1* cKO; *Casq1*^–/–^ (cKO^–/–^) mice. Western blot and quantitative analysis of mRNA level using the heart tissues demonstrated loss of both Znhit1 and Casq1 proteins (Figure 7, A and B). We found that deletion of *Casq1* could postpone the death of *Znhit1* cKO mice by several days, although loss of *Casq1* did not improve the survival of *Znhit1* cKO mice (Figure 7C). However, histological analysis of the heart tissues indicated that deletion of *Casq1* could substantially alleviate the vacuolar formation in P25 *Znhit1* cKO mice (Figure 7, D and E). Then we detected the pattern of Casq1 and SERCA2a in the vacuoles by IF. While Casq1 aggregates disappeared in the vacuoles (Figure 7F), SERCA2a was located around the vacuoles (Figure 7G). These results demonstrate that profoundly enhanced Casq1 is involved in the vacuolar formation observed in the heart of *Znhit1* cKO mice.

## Discussion

In this study, we identified the SRCAP chromatin remodeler as a regulator of cardiac Ca^2+^ homeostasis and that Znhit1 is required for postnatal heart function (Figure 6E). Previously, it was believed that of the 2 Casq isoforms, only Casq2 was present in the heart tissue; hence, Casq2 was called heart specific (39). However, we found that both Casq1 mRNA and protein were detectable at a very low level in the heart. Upon deletion of *Znhit1*, the expression of *Casq1* became prominent with a 10- to 30-fold increase in the heart tissue, indicating a suppression status of *Casq1* by Znhit1 under normal conditions. Both Casq1 and Casq2 showed high homology and both possess strong ability to bind Ca^2+^ in the SR. Massively enhanced Casq1 level in the cardiomyocytes promoted storage of Ca^2+^ in the SR, which disrupted cardiac Ca^2+^homeostasis and was deleterious for heart function. This is supported by findings from a previous study in which overexpression of *Casq2* with 10-fold higher levels in the heart caused severe dilated cardiomyopathy and premature death (6, 7).

Another intriguing finding of this study is idiopathic vacuolar cardiomyopathy observed in the *Znhit1* mutant heart. Vacuolar cardiomyopathy is usually linked to lysosome dysfunction. However, we did not discern the autophagic structure in the vacuoles of *Znhit1* mutant heart by ultrastructural study. Although the typical feature of autophagic vacuolar cardiomyopathy is a deficiency or absence of the lysosomal protein LAMP2, we did not detect a reduced protein level of LAMP2 (data not shown). Thus, the vacuolar cardiomyopathy in the *Znhit1* mutant heart was a type of idiopathic vacuolar cardiomyopathy that did not originate from abnormal lysosomal function. The presence of SERCA2a and Casq1 in the vacuoles indicated that the vacuoles were of SR origin, which was supported by the following evidence: first, transfection of L6 myoblast cells with *Casq1* showed that Casq1 protein tended to aggregate within the endoplasmic reticulum lumen and, in turn, drove vacuolar formation (40). Second, it was reported that in human patients affected by a mild myopathy, an excess of Casq1 protein formed inclusions in the vacuoles; this condition was named Casq storage myopathy (41). Last, in 2 other studies of patients with *Casq1* mutation (Asp244Gly), large SR vacuoles containing characteristic inclusions of Casq1 proteins were reported (22, 23). Our findings help the understanding of the pathogenesis of fatal vacuolar cardiomyopathy that is not associated with lysosomal disorders (12). Casq1 aggregation in the SR might be 1 of the causative conditions for idiopathic vacuolar cardiomyopathy. In addition, we found that deletion of *Casq1* could postpone the death of *Znhit1* cKO mice by several days, although loss of *Casq1* did not improve the survival of *Znhit1* cKO mice. Histological analysis of the heart tissues indicated that deletion of *Casq1* could substantially alleviate the vacuolar formation in *Znhit1* cKO mice. These results demonstrate that profoundly enhanced Casq1 is involved in the vacuolar formation observed in the heart of *Znhit1* cKO mice.

It was reported that 1 of the components of the Mediator complex, MED12, regulates postnatal heart function and deletion of *MED12* causes progressive dilated cardiomyopathy (42). Further study showed that MED12 interacts with the transcription factor of MEF2 and that MED12 and MEF2 co-occupy the promoters of calcium-handling genes. MED12 was identified as a regulator of a network of calcium-handling genes. Two recent studies have demonstrated that TBX5 regulates a network of cardiac channel genes, including *RyR2* and *SERCA2*, to maintain cardiac rhythm, which is involved in atrial fibrillation (43, 44). In addition, the RNA-binding protein RBM20 was also found to play a critical role in postnatal heart function, and RBM20 affects the genes involving calcium handling and muscle contraction (45). Thus, transcriptional regulation of the calcium-handling genes is a common mechanism in modulating postnatal heart function.

In summary, the SRCAP remodeler is a regulator of cardiac Ca^2+^ homeostasis and is required for heart function. Meanwhile, our findings help understand non-lysosomal vacuolar cardiomyopathy.

## Methods

### Mice.

In this study, we used the following mouse strains: *Znhit1* floxed mice (17), *Myh6-Cre* mice (21), and *Casq1*^–/–^ mice (strain T017350; GemPharmatech). All mouse strains were maintained on the C57BL/6 genetic background. Mice were group housed in accordance with the regulations on mouse welfare and ethics of Nanjing University, with 12-hour/12-hour light-dark cycles and had ad libitum access to food and water. Primer sequences for genotyping were as follows: *Znhit1*, 5′-GTTGGGCATCTGCCTTTC-3′ and 5′-CCCTGCCTACATCTGCACTAA-3′; *Cre*, 5′-AATGCTTCTGTCCGTTTGC-3′ and 5′-ACCAGAGTCATCCTTAGCG-3′; *Casq1* primers, 5′-GCATCCCTAATGGAGCTAGGATAC-3′ and 5′-GCGGGTATAGGTTATGAGGACTG-3′; and second 5′-TTCGCTACCTTCGACAGCAAG-3′ and 5′-GATGGTCATAGGCTCTTCCATG-3′.

### Antibodies.

Znhit1 (ab238125), H2A.Z (ab4174), and H3K4me3 (ab1012) were purchased from Abcam (Cambridge); H3K27me3 (9733) was from Cell Signaling Technology. Casq1 (26665-1-AP), Casq2 (18422-1-AP), ATP2A2/SERCA2a (26665-1-AP), for Western blotting, were purchased from Proteintech; ATP2A2/SERCA2a (sc-376235), for IF, was purchased from Santa Cruz; Gapdh Ab (AP0063), goat anti–mouse IgG (H+L)-HRP (BS12478), goat anti–rabbit IgG (H+L)-HRP (BS13278) were purchased from Bioworld; Alexa Fluor 488/Cy3/Cy5–labeled secondary Abs (115-514-166,112-165-167, and 711-175-152) were purchased from Jackson Immunoresearch.

### Histological analysis and IF staining.

Mice were euthanized by cervical dislocation and heart tissues were collected quickly; blood cells were washed out in cold PBS. For paraffin sections, the hearts were fixed in 4% paraformaldehyde overnight at 4°C. Fixed hearts were washed in cold PBS for 30 minutes, dehydrated in gradient ethanol (1 hour each at 35%, 50%, 75%, 85%, and 95%), rendered transparent 3 times in *n*-butanol for 1 hour each, incubated 3 times in 65°C paraffin, embedded in paraffin, and then sectioned at a thickness of 7 um (46). For frozen sections, the hearts were fixed for 2 hours in 4% paraformaldehyde on ice, then transfected into 30% sucrose overnight at 4°C, frozen in OCT compound (Thermo Fisher Scientific, catalog Pink Neg-50 no. 6502P), and sectioned at 10-um thickness.

Paraffin sections were used for H&E staining and Masson’s trichrome staining, as previously described (20, 21). Paraffin sections and frozen sections were both used for IF staining. IF staining was performed according to the standard protocols, as previous described (46). For WGA and DAPI staining, paraffin sections were dewaxed and rehydrated, rinsed 3 times in PBS, incubated with WGA and DAPI at room temperature for 1 hour. Afterward, the sections were rinsed 3 times in PBS and mounted in 50% triglyceride. Images for IF staining were captured using an Olympus FV3000 confocal microscope.

### Echo and ECG of mouse heart.

Mice were anesthetized through continuous inhalation of gaseous isoflurane and their heart rate was maintained between 350 and 450 beats/min. Mouse body temperature was monitored with a rectal thermometer and maintained between 36°C and 38°C. Noninvasive transthoracic Echo was tested with the Vevo770 UBM system (Visual Sonics), which was equipped with M model, and 2-dimensional measurements were recorded. Left ventricular end diastolic diameter and left ventricular end systolic diameter were counted. Left ventricular ejection fraction and left ventricular fraction shortening were calculated according to the user guidelines of the Vevo770 UBM system.

### TUNEL staining.

TUNEL staining was performed as previously described (47). Briefly, paraffin-embedded sections were dewaxed, rehydrated, and treated with 20 mg/mL proteinase K for 20 minutes. Afterward, TUNEL staining was performed according to the user manual (Vazyme, A113). In each experiment, negative control, positive control, and experimental groups were performed at the same time.

### RNA isolation, real-time qPCR and RNA-Seq analysis.

Mouse hearts (atrial tissues were removed) were snap frozen and stored at –80°C until use. Total RNA was isolated using TRIzol regents (Life Technologies) according to the standard user manual. Obtained total RNA was reverse transcribed into cDNA using the HiScript II 1st Strand cDNA Synthesis kit (Vazyme, R211-02). qRT-PCR was performed with an ABI QuantStudio 5 Real-Time PCR System (Applied Biosystems) using AceQ qPCR SYBR Green Master Mix (Vazyme, Q141-03) as the fluorescent dyes. Primer sequences are listed in Supplemental Table 2.

For RNA-Seq analysis, 3 biological replicates in each group (i.e., the control group and *Znhit1*-deficient group) were assessed with the Illumina HiSeq xten/NovaSeq 6000 sequencer by Shanghai Majorbio Bio-pharm Technology Co., Ltd. The raw paired-end RNA-Seq reads were aligned to the mouse GRCm38 reference genome with STAR (version 2.7.1a) (49). Cuffnorm (version 2.2.1) was used to obtain expression matrix of difference samples of fragments per kilobase per million reads. Differentially expressed gene expression analysis was performed by Cuffdiff2, version 2.2.1 (49). Heatmaps of differentially expressed gene expression were generated with R package pheatmap (version 1.0.10; (https://cran.rproject.org/web/packages/pheatmap/index.html). The analysis of GO and Kyoto Encyclopedia of Genes and Genomes pathways was done using R package clusterProfiler, version 4.0.5) (50).

### Western blotting analysis.

Mouse hearts (atrial tissues were removed) were snap frozen in liquid nitrogen and stored at –80°C until use. Tissue were homogenized in RIPA buffer (Beyotime Biotechnology, P0013B) supplemented with PMSF and protease inhibitor cocktails (Roche, 11873580001). Equal amounts of sample proteins were loaded onto SDS polyacrylamide gels, separated by a vertical electrophoresis system, and finally transferred to a PVDF membrane (EMD Millipore). Afterward, the membranes were blocked in 5% BSA in Tris-buffered saline with Tween detergent (TBST; 150 mM NaCl, 50 mM Tris, and 0.5 nM Tween 20 at pH 7.5) for 1 hour and incubated with primary Abs in 5% BSA overnight at 4°C. The following day, the membranes were rinsed 3 times in TBST for 5 minutes each, incubated with secondary Abs at room temperature for 2 hours, rinsed 3 times in TBST, and finally visualized using ECL. Quantification analysis of protein levels was performed using ImageJ software (NIH).

### Calcium imaging.

The isolation and culture of postnatal mouse cardiomyocytes were performed according to the previously described protocol (51). Isolated cardiomyocytes were seeded in glass-bottomed, 35-mm confocal dishes. For calcium imaging, the cardiomyocytes were incubated in 5 mM Fluo-4, AM (Thermo Fisher Scientific, F14201) of culture medium for 30 minutes at room temperature, washed 3 times with Tyrode’s salt solution (140 mM NaCl, 5 mM KCl, 2 mM MgCl_2_, 10 mM glucose, 10 mM HEPES, 1.8 mM CaCl_2_, pH 7.4) and incubated in Tyrode’s salt solution for 15 minutes. Line-scan images were performed with an Olympus FV3000 confocal microscope. A final concentration of 10 mM caffeine was added into the dishes to induce SR calcium release, and the release signal was recorded.

### Transmission electron microscopy.

Mice were euthanized by cervical dislocation. Heart was quickly collected, blood cells were washed out in cold PBS and immersed in cold 2.5% glutaraldehyde in 0.1 M sodium phosphate (pH 7.4). A pre-cold sharp blade was used to cut a piece of tissue from the left ventricular apex and then cut it into smaller pieces (<2 mm^3^) in cold 2.5% glutaraldehyde. Small pieces of cardiac tissue were fixed overnight at 4°C using 2.5% glutaraldehyde. To preserve the structural integrity of tissue, the whole process was completed quickly. The following procedures were conducted in the electron microscopy center of Zhejiang University.

### CUT&Tag analysis.

Fresh mouse heart tissues (atrial tissues were removed) were used for extracting nuclei with a nuclear extraction kit (Solarbio, SN0020). Approximately 10,000 cell nuclei were prepared for CUT&Tag and library construction as previously described (36). The reagents used in experiments all came from the Hyperactive In-Situ ChIP Library Prep Kit for Illumina (pA-Tn5; Vazyme, TD902-02) and the TurePrep Index Kit V2 for Illumina (Vazyme, TD202). All experimental procedures were performed according to the Vazyme user manual. Afterward, the sequence and bioinformatic analyses were conducted by the Novogene Bioinformatic Institute. In brief, CUT&Tag libraries were sequenced with the Illumina Nova6000 sequencer. Sequencing reads were aligned to the mouse mm10 reference genome with Bowtie2 (version 2.4.4) (http://bowtie-bio.sourceforge.net/bowtie2/index.shtml). Duplicated reads were removed with Sambamba markup (0.8.1) (http://lomereiter.github.io/sambamba/). MACS2 (version 2.2.7.1) (https://pypi.python.org/pypi/MACS2) was used to call peaks (52). Peak annotation was performed by R package ChIPseeker (version 1.28.3) (53).

The RNA-Seq and CUT&Tag data have been deposited in the National Center for Biotechnology Information’s Gene Expression Omnibus (accession no. GSE194164).

### Data and materials availability.

All data are available in the main text or the Supplemental materials.

### Statistics.

All experiments were done with at least 3 biological repeats. Data are reported as mean ± SD. Statistical results were performed with GraphPad Prism, version 8.0, software. For comparison of 2 groups, statistical significance was determined by unpaired, 2-tailed Student’s *t* tests. *P* < 0.05 was considered significant, *P* < 0.01 was considered highly significant, and *P* < 0.001 was considered extremely significant.

### Study approval.

The IACUC of the Model Animal Research Center of Nanjing University approved all animal procedures used in this study.

## Author contributions

YC and ZY conceived of the study and wrote, reviewed, and edited the manuscript. MX and WZ conducted the experiments. YC and WF investigated the results. XL maintained the mouse line. ZY supervised the study. YC wrote the original draft.

## Supplementary Material

Supplemental data

## Version 1 02/15/2022

In-Press Preview

## Version 2 03/22/2022

Electronic publication
