# Supplementary material for: Critical role of Znhit1 for postnatal heart function and vacuolar cardiomyopathy
Source: JCI Insight. 2022 Mar 22;7(6):e148752. doi: 10.1172/jci.insight.148752 (PMC8986070; doi:10.1172/jci.insight.148752)
Supplement: Supplemental data [file jciinsight-7-148752-s197.pdf]

Critical role of Znhit1 for post-natal heart function and vacuolar cardiomyopathy

Yingchao Shi[1], Wenli Fan[1], Mingjie Xu[1], Xinhua Lin[2], Wukui Zhao[1]* and Zhongzhou Yang[1]*

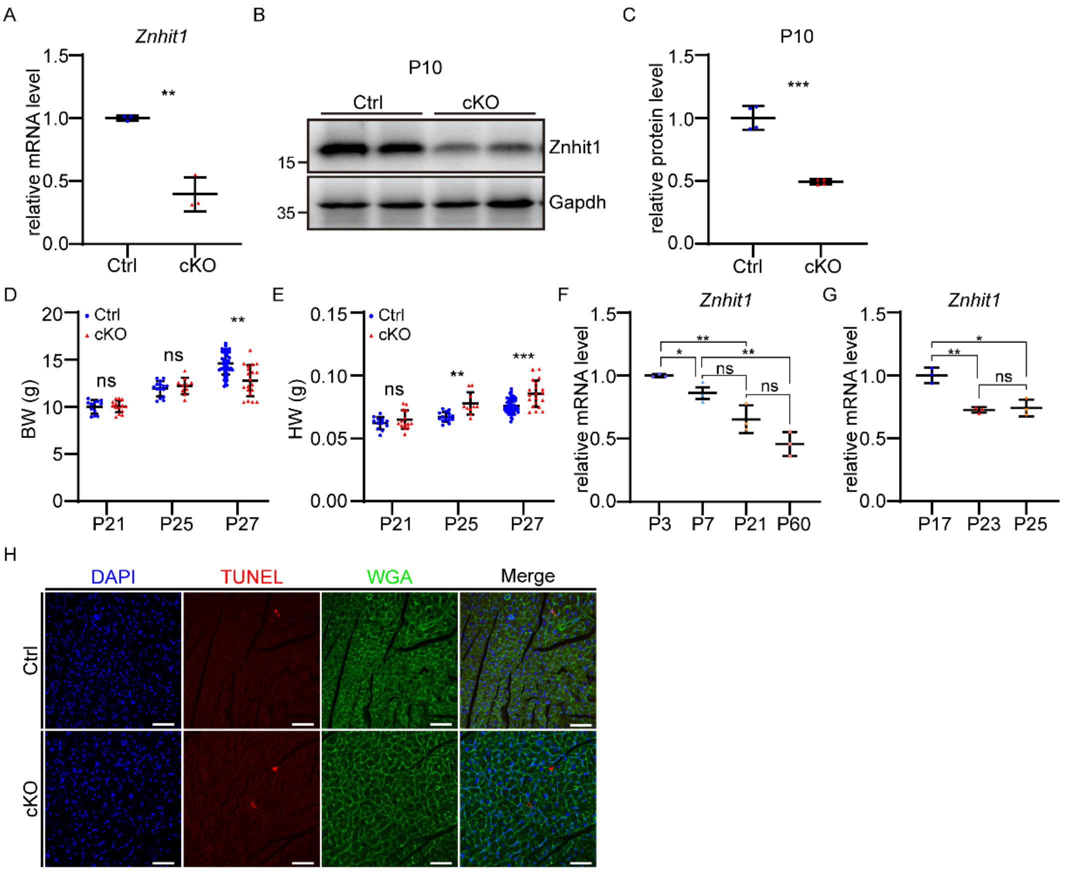

Figure S1. Conditional knockout of *Znhit1* in postnatal mouse heart.

(A) Expression level of *Znhit1* mRNA in control (Ctrl) and *Znhit1* conditional KO (cKO) hearts at P10. N=3 for each group. **(B** and **C)** Western blotting and quantitative analysis of Znhit1 protein level in Ctrl and cKO hearts at P10. Gapdh was used as loading control. **(D** and **E)** Body weight and heart weight of mice at P21, P25 and P27. Ctrl, P21 (n=13); P25 (n=15); P27 (n=52). cKO, P21 (n=14); P25 (n=10); P27 (n=20)**. (F** and **G)** mRNA expression patterns of *Znhit1* in postnatal mouse hearts. N=3 for each group. **(F)** P3 to P60 and **(G)** 17 to P25. **(H)** Representative TUNEL staining at P25 and P27. Scale bar, 50 μm. Control (Ctrl) mice were *Znhit1* $^{f/f}$ or $^{f/+}$ littermates. Data are presented as mean ±SD. ns (not significant), *p < 0.05, **p < 0.01, and ***p < 0.001 by unpaired two-tailed Student's t-test.

A

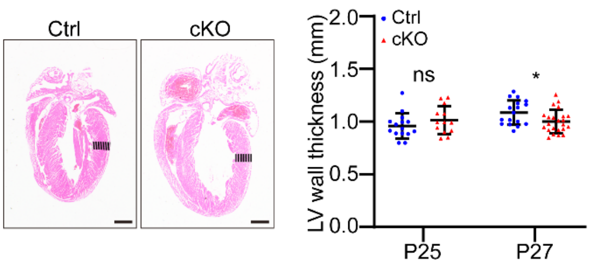

Figure S2. Histological analysis of the left ventricular wall thickness.

(A) The left panel shows histological study of the heart and the right panel shows quantitative analysis of the thickness of the left ventricular wall. Ctrl, P25 (n=15); P27 (n=13). cKO, P25 (n=17); P27 (n=23). Control (Ctrl) mice were *Znhit1* $^{f/f}$ or $^{f/+}$ littermates. Data are presented as mean±SD. ns (not significant) and $*p < 0.01$ by unpaired two-tailed Student's t-test.

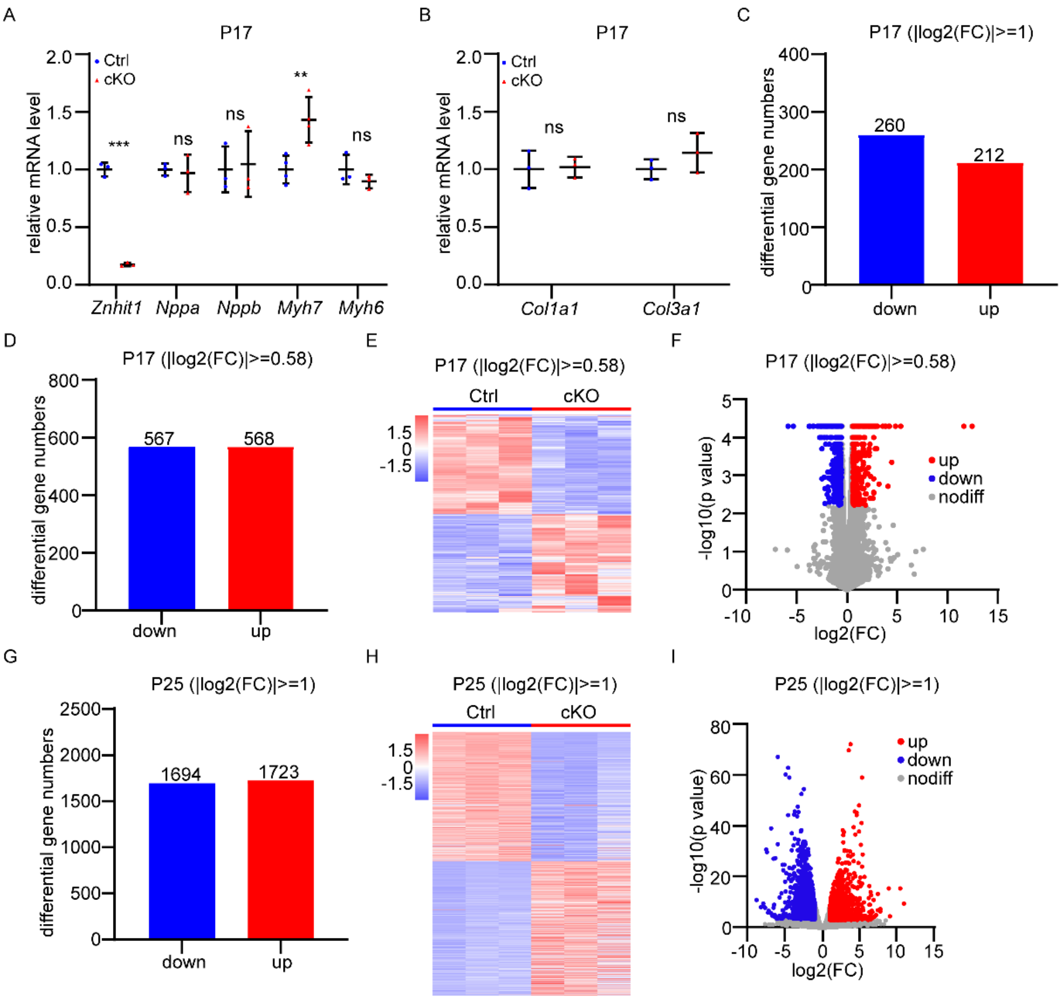

Figure S3. Gene expression and transcriptomic analysis.

(A) qRT-PCR analysis of cardiac hypertrophic marker genes at P17. N=3 for each group. **(B)** mRNA expression levels of fibrosis-related genes at P17. N=3 for each group. **(C)** Numbers of differential genes (| log2(fold change) | ≥ 1 and *P* value ≤ 0.05) at P17. **(D)** Numbers of differential genes (| log2(fold change) | ≥ 0.58 and *P* value ≤ 0.05) at P17. **(E** and **F)** Heatmap and volcano plot. **(G)** Numbers of differential genes (| log2(fold change) | ≥ 1 and *P* value ≤ 0.05) at P25. **(H** and **I)** Heatmap and volcano plot. Control (Ctrl) mice were *Znhit1* $^{f/f}$ or $^{f/+}$ littermates. Data are presented as mean± SD. ns (not significant), **$p < 0.01$, and ***$p < 0.001$ by unpaired two-tailed Student's t-test.

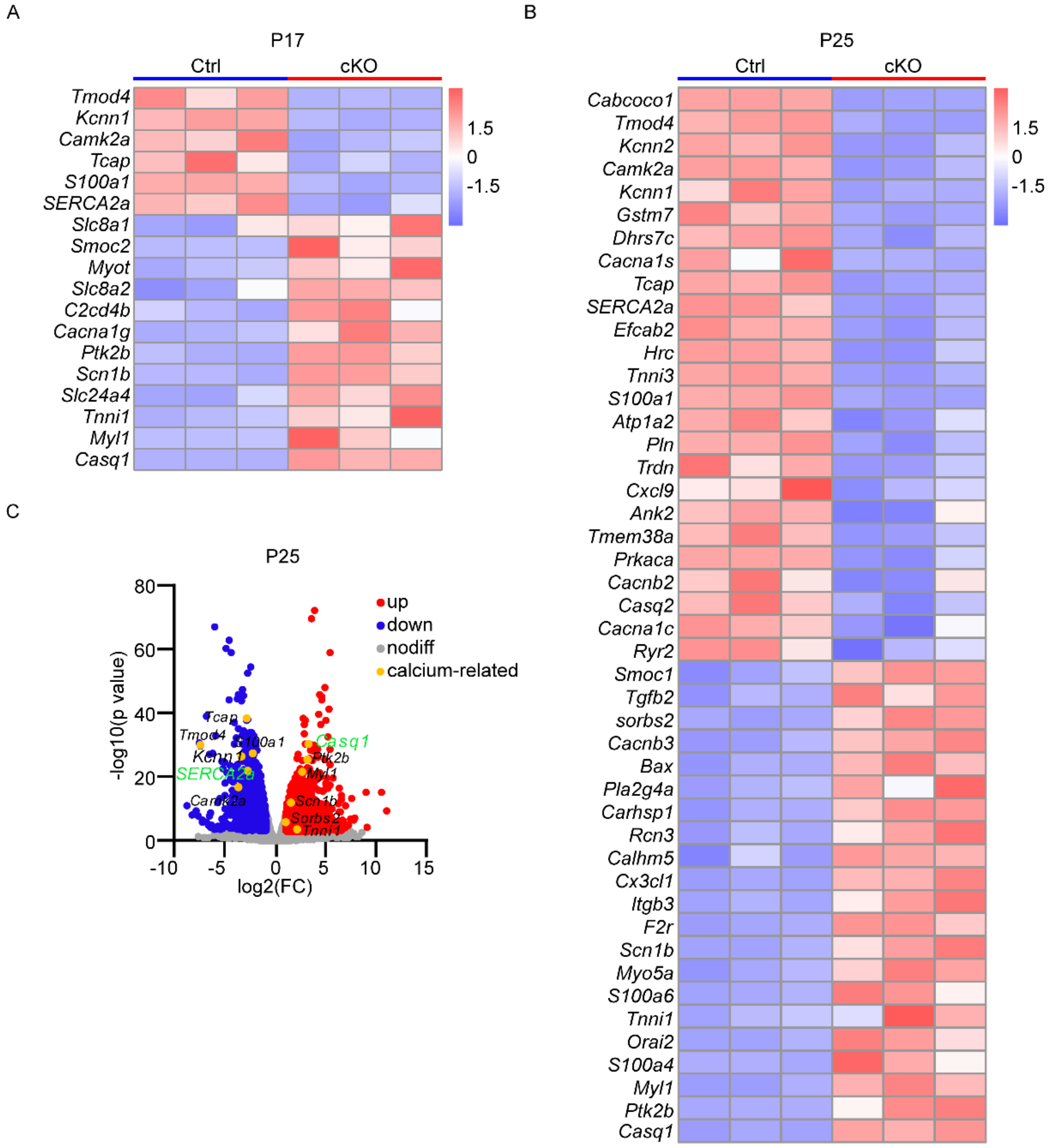

Figure S4. Enrichment analysis of Ca^{2+} handling genes.

(A and B) Heatmap analysis of differential genes at P17 and P25. **(C)** Volcano plot analysis of 12 common changed Ca^{2+} handling genes at P25. Control (Ctrl) mice were *Znhit1* $^{f/f}$ or $^{f/+}$ littermates.

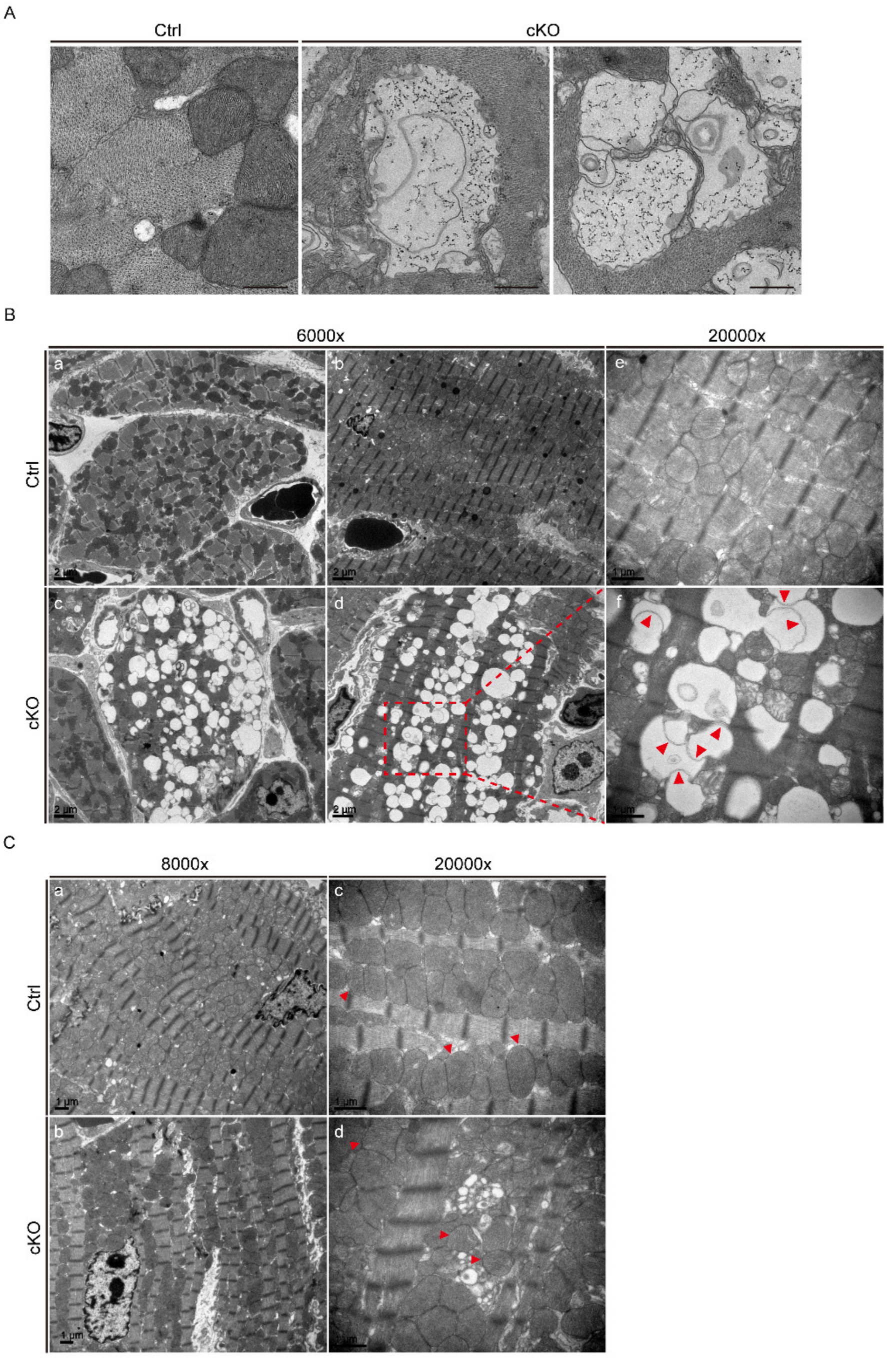

Figure S5. Transmission electron microscopy imaging of the vacuole-like cardiomyocytes.

(A) Transmission electron microscopy imaging for vacuoles containing inclusions**.** 6000 X magnification. Scale bars, 500 nm. **(B)** Transmission electron microscopy imaging under higher magnification. (a, b, c, d), 6000 X magnification. Scale bars, 2 μm; (e, f), 20000 X magnification. Scale bars, 1 μm. Red arrowheads indicated the membrane structure around vacuoles and swollen mitochondria. **(C)** Transmission electron microscopy imaging of the non-vacuole-like cardiomyocytes under higher magnification. (a, b), 8000 X magnification. Scale bars, 1 μm. (c, d), 20000 X magnification. Scale bars, 1 μm. Red arrowheads indicated the normal mitochondrial structure. Control (Ctrl) mice were *Znhit1* $^{f/f}$ or $^{f/+}$ littermates.

A

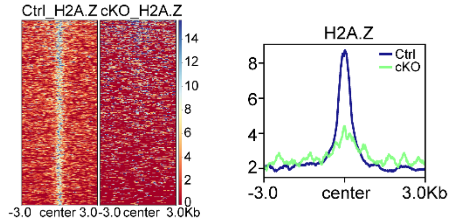

B

Down-regulated genes

ID	pathway
mmu00280	Valine,leucine and isoleucine degradation
mmu01212	Fatty acid metabolism
mmu04260	Cardiac muscle contraction
mmu05414	Dilated cardiomyopathy
mmu05412	Arrhythmogenic RV cardiomyopathy

Up-regulated genes

ID	pathway
mmu04151	PI3K-Akt signaling pathway
mmu04810	Regulation of actin cytoskeleton
mmu04630	JAK-STAT signaling pathway
mmu04060	Cytokine-cytokine receptor interaction
mmu05206	Micro RNAs in cancer

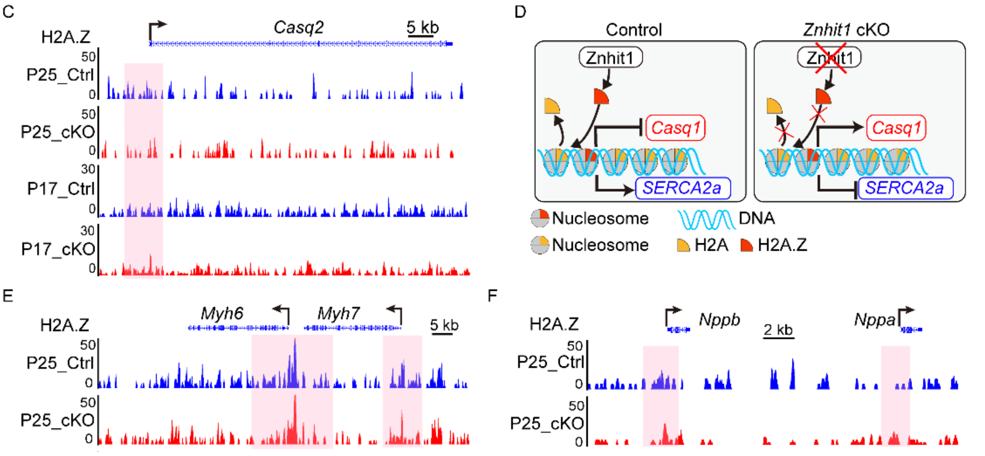

Figure S6. The loss of Znhit1 impaired the distribution of H2A.Z on genome.
(A) Heatmap and metaplot representing CUT&Tag intensities ±3 kb around the center of H2A.Z target loci of the 539 genes. **(B)** KEGG pathway analysis of the upregulated and downregulated genes (539). **(C)** The distribution of H2A.Z at the promoter of *Casq2*. **(D)** The model of Znhit1 regulating the expression of *SERCA2a* and *Casq1*. **(E** and **F)** The distribution of H2A.Z at the promoters of *Myh6*, *Myh7*, *Nppb* and *Nppb*. Control (Ctrl) mice were *Znhit1* $^{f/f}$ or $^{f/+}$ littermates.

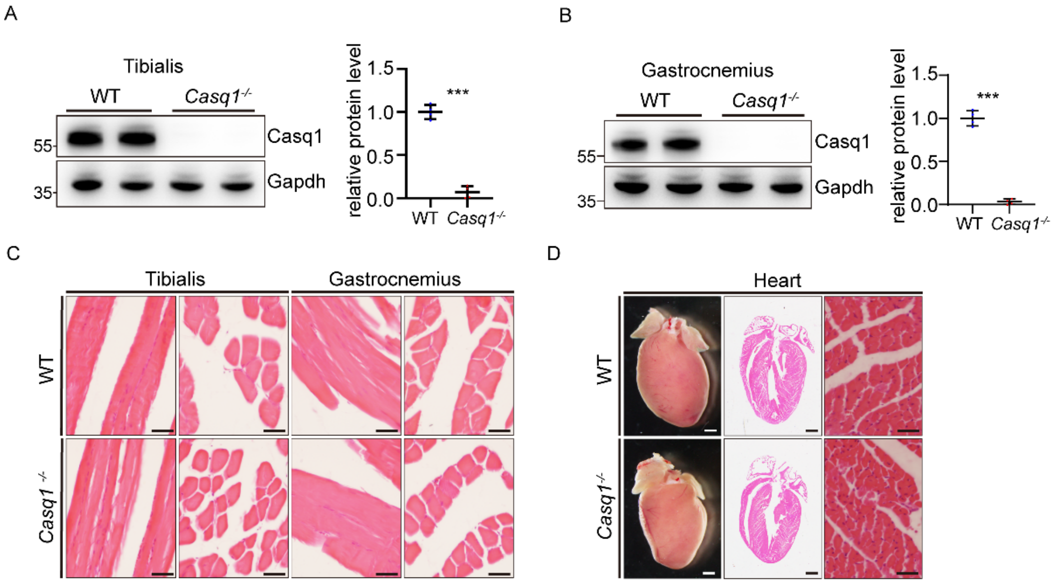

Figure S7. The loss of Casq1 did not cause obvious pathological disorders in whole body.

(A and **B)** Western blotting and quantitative analysis for Casq1 level in tibialis anterior muscle and gastrocnemius muscle of *Casq1*$^{-/-}$ mice. WT, wild type. Gapdh was used as a loading control. **(C)** Representative images of HE staining for tibialis anterior muscle and gastrocnemius muscle. WT, wild type. 40 X magnification. Scale bar, 20 μm. **(D)** Representative pathological analysis for hearts of *Casq1*$^{-/-}$ mice. WT, wild type. Scale bar, 1 mm, 1 mm and 20 μm, respectively. Control (Ctrl) mice were *Znhit1* $^{f/f}$ or $^{f/+}$ littermates. Data are presented as mean ± SD. ****p* < 0.001 by unpaired two-tailed Student's t-test.

Table S1. Non-standard Abbreviations and Acronyms

Znhit1	Zinc Finger HIT-Type Containing 1
SRCAP	Snf2 Related CREBBP Activator Protein
H2A.Z	H2A histone family member Z
SR	Sarcoplasmic reticulum
ECC	Excitation-contraction coupling
EF	Ejection fraction
FS	Fractional shortening
LVID; d	Left ventricular internal dimension at diastole
LV vol; d	Left ventricular volume at diastole
RyR2	Ryanodine receptor 2
Pln/Plb	phospholamban
Atp2a2/SERCA2a	Sarcoplasmic/endoplasmic reticulum calcium ATPase 2a
Casq	Calsequestrin
Nppa/ANF	Natriuretic peptide type A
Nppb/BNP	Natriuretic peptide type B
Myh6	Myosin, heavy polypeptide 6, cardiac muscle, alpha
Myh7	Myosin, heavy polypeptide 7, cardiac muscle, alpha
Col1a1	Collagen type I alpha 1 chain
Col3a1	Collagen type III alpha 1 chain
Col5a1	Collagen type V alpha 1 chain
Col8a1	Collagen type VIII alpha 1 chain

Table S2. Primers for qRT-PCR

Gene	Forward	Reverse
Mouse *Gapdh*	5'-AGGTCGGTGTGAACGGATTTG-3'	5'-TGTAGACCATGTAGTTGAGGTCA-3'
Mouse *Znhti1*	5'-CAGACGGCGAGACAAGTTC-3'	5'-CAAACTGAGGTAGCCTCTT-3'
Mouse *Col1a1*	5'-GCTCCTCTTAGGGGCC ACT-3'	5'-CCACGTCTCACCATTGGGG-3'
Mouse *Col3a1*	5'-CCTGGCTCAAATGGCTCAC-3'	5'-CAGGACTGCCGTTATTCCCG-3'
Mouse *Col5a1*	5'-CTTCGCCGCTACTCCTGTTC-3'	5'-CCCTGAGGGCAAATTGTGA-3'
Mouse *Col8a1*	5'-ACTCTGTCAGACTCATTCAGG-3'	5'-CAAAGGCATGTGAGGGACT-3'
Mouse *Nppa*	5'-GCTTCCAGGCCATATTGGAG-3'	5'-GGGGGCATGACCTCATCTT-3'
Mouse *Nppb*	5'-GAGGTCACTCCTATCCTCTGG-3'	5'-GCCATTTCCTCCGACTTTTCTC-3'
Mouse *Myh7*	5' -ACTGTCAACACTAAGAGGGTCA-3'	5'-TTGGATGATTTGATCTTCCAGGG-3'
Mouse *Myh6*	5'-GCCCAGTACCTCCGAAAGTC-3'	5'-GCCTTAACATACTCCTCCTTGTC-3'
Mouse *SERCA2a*	5'-ACTTCTTGATCCTCTACGTG-3'	5'-AAATGGTTTAGGAAGCGGTT-3'
Mouse *Casq1*	5'-CTGGCACTGCTGTTTGTACTG-3'	5'-GGGGGCTCATGGTAGAGGAG-3'
Mouse *Casq2*	5'-CTCTATTACCACGAACCTGTGTC-3'	5'-GGCCACAAGCTCCAGTACAAT-3'
Mouse *RyR2*	5'-ATGGCTTTAAGGCACAGCG-3'	5'-CAGAGCCCGAATCATCCAGC-3'
Mouse *Pln*	5'-CCTTCCTGGCATAATGGAAA-3'	5'-CATGTTGCAGGTCTGGAGTG-3'

Table S3. FPKM value of Calcium regulated genes of P17 RNA-seq results.

Gene	Ctrl_FPKM	cKO_FPKM	Fold change	P value
Tmod4	5.81318	0.833696	0.119832	0.00005
Camk2a	10.32008	6.05417	0.58664	0.00005
Kcnn1	1.461967	0.256211	0.175251	0.00005
Tcap	651.618	386.61	0.593308	0.00005
SERCA2a	1338.877	809.6423	0.604718	0.00225
S100a1	334.6537	200.937	0.600433	0.00005
Pln	1798.09	1616.777	0.899163	0.164994
Casq2	267.5463	223.28	0.834547	0.161788
RyR2	49.9331	47.67157	0.954709	0.770969
Slc8a1	7.10236	11.7612	1.655957	0.00005
Sorbs2	75.9807	117.773	1.550038	0.0001
Scn1b	7.11558	30.15207	4.237471	0.00005
Tnni1	0.793249	4.95782	6.25002	0.00005
Myl1	15.1668	102.8336	6.780176	0.00005
Ptk2b	0.768711	2.942423	3.827737	0.00005
Casq1	7.29955	53.03007	7.264841	0.00005

Table S4. FPKM value of Calcium regulated genes of P25 RNA-seq results

Gene	Ctrl_FPKM	cKO_FPKM	Fold change	P value
Tmod4	11.32333	0.383333	0.033853	9.12E-46
Camk2a	12.58333	1.296667	0.103046	4.84E-16
Kcnn1	4.583333	0.49	0.106909	1.7E-30
Tcap	1283.263	218.7333	0.170451	1.41E-40
SERCA2a	1893.297	341.7767	0.180519	5.45E-22
S100a1	860.1067	224.1867	0.26065	1.07E-29
Pln	2585.26	834.3733	0.322743	5.15E-14
Casq2	425.4	207.9	0.488717	1.46E-10
RyR2	54.47667	33.69	0.61843	2.73E-05
Slc8a1	18.55333	42.51667	2.291592	0.082458
Sorbs2	57.07667	153.8133	2.694855	1.62E-06
Scn1b	14.37	51.96667	3.61633	2.21E-13
Tnni1	1.02	5.246667	5.143791	0.000423
Myl1	50.34	375.7967	7.46517	8.52E-22
Ptk2b	2.126667	22.33333	10.50157	8.31E-26
Casq1	19.12667	227.84	11.91216	2.06E-30
